# Next-generation sequencing yields mitochondrial genome of *Coccidophilus cariba* Gordon (Coleoptera: Coccinellidae) from museum specimen

**DOI:** 10.1080/23802359.2019.1679684

**Published:** 2019-10-25

**Authors:** Romain Nattier, Karen Salazar

**Affiliations:** a Institut de Systématique, Evolution, Biodiversité, ISYEB, Muséum national d'Histoire naturelle (MNHN), CNRS, Sorbonne Université, EPHE, Université des Antilles, Paris, France;; b Grupo de Inverstigación Insectos de Colombia, Instituto de Ciencias Naturales, Universidad Nacional de Colombia, Bogotá, Colombia

**Keywords:** Illumina, ladybird, Microweiseinae, mitogenome, phylogeny

## Abstract

We assembled and annotated the first complete mitochondrial genome of a species from the subfamily Microweiseinae, *Coccidophilus cariba* Gordon, a predator of scale insect pest. The circular mitogenome consists of 15,343 bp in length, including 13 PCGs, 22 tRNA, and 2 rRNA genes, and exhibits the typical ladybird mitogenome structure. A phylogenetic analysis with the published mitogenomes of other 12 ladybirds is presented, which confirms the position of Microweiseinae as a sister group of Coccinellinae. The *C. cariba* mitogenome could be a useful source to future studies in the relationships inside the subfamily and the genus.

Coccinellidae comprises around 360 genera and 6000 species (Vandenberg 2002) and has been recently divided into the subfamilies Microweiseinae and Coccinellinae (Seago et al. 2011). To date, 28 mitochondrial genomes, complete or partial have been published in Genbank, all belonging to the Coccinellinae. The Microweiseinae *Coccidophilus cariba* Gordon is a scale predator and is the only member of *Coccidophilus* living in the West Indies (Gordon 1978).

The specimen sequenced was collected in Guaeloupe in 1997 (Ravine Chaude, GP 1417) and a voucher was deposited in the MNHN (MNHN-EC-7788). DNA was extracted from the whole specimen, following the non-destructive protocol described in Gilbert et al. (2007), and then quantified using a Qubit fluorometer (Life Technologies, Paisley, UK). Genomic DNA was indexed and libraries prepared using the NEBNext Ultra II library prep kit (New England BioLabs; NEB), with a modified version of Meyer and Kircher (2010) protocol. DNA was sequenced at the Genome and Transcriptome Platform of Toulouse (Genotoul, France) using Illumina HiSeq 3000 technology (150 bp paired‐end). Quality and length distribution of the sequences were inspected prior and post-clean using FASTQC (Andrews 2010). Low-quality reads and adaptor contamination were then trimmed using the BBDuk plugin as implemented in Geneious prime 2019.1.3 (Biomatters Ltd., Auckland, New Zealand). To separate the mitochondrial genome sequences from the rest of the sequence data, an iterative read mapping strategy was employed using Geneious workflow Align/Assemble ‘Map to Reference’ and the mitochondrial sequence of *Henosepilachna pusillanima* as reference (Geneious mapper, custom sensitivity with a maximum mismatch of 30%, fine-tuning 25 times). We repeat this operation with a mismatch of 10% and we conclude with a final de novo step (maximum mismatch of 30%). 7,514 overlapped reads (mean 109.1 bp) were used to generate a consensus sequence and to create a circular molecule. We obtain a mitogenome of 15,343 bp long, with a read coverage of 53×. The identity and position of 13 PCGs, 22 tRNA, and 2 rRNA genes were determined using the MITOS web server (Bernt et al. 2013; http://mitos.bioinf.uni-leipzig.de), in combination with the annotation with the mitogenome of *Henosepilachna pusillanima* in Geneious. The gene order is similar to other Coccinellidae mitochondrial genomes. The mitochondrial genome was submitted to GenBank (accession number # MN447521) with the submission tool implemented in Geneious.

The phylogenetic position of *C. cariba* was inferred from 12 mitochondrial genome sequences of Coccinellidae available in Genbank, with *Priasilpha obscura* (Priasilphidae) and *Cucujus clavipes* (Cucujidae) as outgroups. The 13 PCGs and the 2 rRNA was extracted from each genome, aligned separately with the MAFFT algorithm (Katoh and Standley 2013) implemented in MEGA version X (Kumar et al. 2018), then concatenated. Phylogenetic analysis was performed with using Iq-Tree 1.5 (Nguyen et al. 2015) as implemented in the W-Iq-Tree web server (http://iqtree.cibiv.univie.ac.at; Trifinopoulos et al. 2016). All PCGs and rRNA alignment were partitioned, the models of substitution were automatically selected, and an ultrafast bootstrap with 1,000 iterations was performed. The resulting topology (Figure 1) confirms the position of Microweiseinae as sister group of Coccinellinae (Seago et al. 2011; Robertson et al. 2015).

**Figure 1.**
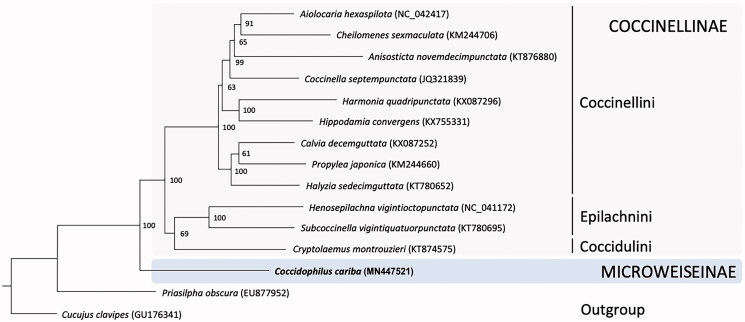
Phylogenetic relationships of Coccinellidae, based on 13 mitochondrial genomes, reconstructed with Iq-Tree 1.5 as implemented on the W-Iq-Tree web server. Ultrafast bootstrap support from 1000 replicates is indicated at the right of each node. For each species is showed the GenBank accession number of the mitochondrial genome.
